# Preservation of Soil Ruins After High Temperatures: Water Absorption and Compressive Strength

**DOI:** 10.3390/polym17050656

**Published:** 2025-02-28

**Authors:** Miao Li, Yifan Zhang, Zhilin Du, Yao Zheng, Senbiao Liu, Hongjie Luo, Waraz Abdul, Jianfeng Zhu

**Affiliations:** Shaanxi Key Laboratory of Green Preparation and Functionalization for Inorganic Materials, Institute of Silicate Cultural Heritage, School of Material Science and Engineering, Shaanxi University of Science and Technology, Xi’an 710021, China

**Keywords:** hydrogel, response surface methodology, soil ruin reinforcement

## Abstract

Soil ruins hold historical significance and serve as witnesses to past civilizations. High temperatures cause soil moisture to evaporate and soil to shrink, leading to cracking issues and making preservation a global challenge. P(AA-AM-AMPS) (Acrylic acid -Acrylamide-2-Acrylamide-2-methy-propenesulfonic acid) composites solve this problem by combining absorbent materials with loess. In this study, P(AA-AM-AMPS) was prepared using a solution method. The water absorption, water retention, air permeability, and compressive strength of P(AA-AM-AMPS) in loess were studied. The results show that after adding P(AA-AM-AMPS) to loess, there is no obvious color difference compared with the blank sample, and the air permeability of the reinforced loess is basically the same as that of the blank soil. After aging resistance tests, the surface cracking of soil clods is significantly reduced compared to that of the blank samples, and their compressive strength improved from 1.8 N/mm^2^ to 2.6 N/mm^2^.

## 1. Introduction

Soil-based ruins are found worldwide and embody the history and civilization of ancient societies; however, their long-term exposure to outdoor conditions has complicated their preservation due to a series of issues resulting from the change from extremely dry and hot weather to wet and cold climates. Thus, identifying the most effective conservation methods has become an urgent priority for researchers [1,2,3,4]. Regarding the reinforcement of soil ruins, there are two methods: physical and chemical. The physical method primarily aims to address collapse issues that affect the stability of the ruins, while the chemical method mainly deals with surface weathering, surface cracks, and other issues [5,6,7,8]. Three types of materials are used in the chemical method: inorganic materials, organic materials, and inorganic-organic materials [9,10,11].

With the development and progress of human society, polymeric materials, as important functional materials, have received increasing attention in the fields of medicine [12,13,14,15], hygiene, agriculture, environmental protection, and personal care [16]. Hydrogels, a type of polymeric material, possess excellent water absorption, water retention, and stability, thus having broad application prospects in various fields [17,18]. The first generation of hydrogels consists mainly of three types: polymers formed through radical chain-induced addition reactions of olefinic monomers, covalently cross-linked hydrophilic polymers, and cellulose hydrogels. Second-generation hydrogels are mainly PEG (polyethylene glycol)/polyester block copolymers. Third-generation hydrogels are formed through stereo complexation, inclusion compounds, metal-ligand coordination, and synthetic peptide chains. Despite several studies on the preparation of hydrogels, there is still a lack of scientific evidence regarding the effects of the degree of neutralization, monomer ratio, and amount of crosslinking agents on the water absorption properties of hydrogels.

Acrylic acid (AA) is used as a polymer monomer in hydrogel preparation due to the presence of its hydrophilic carboxyl group (-COOH) [19,20], which allows it to absorb water tens of times its weight and maintain a stable gel state after absorption [21]. Acrylic acid hydrogels have excellent transparency, flexibility, and weather resistance; however, their poor adhesion limits their further applications. Acrylamide (AM) is a monomer in hydrogels, and its polymers are used in the medical field due to their excellent biocompatibility [22] and non-toxicity. Moreover, acrylamide has strong adhesion properties, enabling good adhesion to sample surfaces. 2-Acrylamido-2-methy-l-propanesulfonic acid is a multifunctional water-soluble anionic surfactant monomer with polymerizable vinyl groups and hydrophilic sulfonic acid groups, which can copolymerize with water-soluble monomers such as acrylonitrile and acrylamide, as well as non-water-soluble monomers such as styrene and vinyl chloride. The introduction of hydrophilic sulfonic acid groups into polymers confers moisture absorption, water permeability, and conductivity to fibers and films, making them commonly used modifiers [23].

The preparation of hydrogels at room temperature can facilitate soil ruin reinforcement. Room-temperature hydrogel preparation primarily generates radicals through oxidation-reduction reactions [24]. The addition of a reductant to peroxides reduces the activation energy required for the generation of radicals, thus increasing the initiation rate or reducing the polymerization temperature. These radicals can initiate the opening of unsaturated bonds, leading to chain growth. The entire polymerization process involves radical generation, chain initiation, and chain termination. Li et al., [25] successfully prepared poly (acrylic acid-acrylamide) copolymers using solution polymerization under constant temperature conditions (45–50 °C) with aluminum hydroxide as the crosslinking agent, achieving a water absorption ratio of 1050 g/g. This demonstrates the excellent water absorption capacity of the hydrogels. However, its high-temperature preparation is not suitable for soil ruins reinforcement applications. Therefore, this study adopts room-temperature hydrogel preparation to lay a solid foundation for field testing.

## 2. Experimental Section

Acrylic acid was obtained from Shanghai, China Aladdin Biochemical Technology Co., Ltd. Acrylamide was obtained from Tianjin, China Dacat Chemical Reagent Factory; 2methyl-acrylamide-2methyl-propanesulfonic acid from shanghai, China Bide Pharmaceuticals; ammonium persulfate from Tianjin, China Tianli Chemical Reagent Co.; and sodium sulfite from Tianjin, China Tianli Chemical Reagent Co. *N*-*N*’-methylene bis-acrylamide was obtained from Tianjin, China Comio Chemical Reagent Co., Ltd. Sodium hydroxide was obtained from Tianjin, China Damao Chemical Reagent Factory.

### 2.1. Synthesis of P(AA-co-AM-co-AMPS)

Acrylic acid (8.71–13.07 g) is added to a beaker and neutralized with NaOH solution with a concentration of 8 mol/L to obtain a mixture with a neutralization of 50%, 60%, 70%, 80%, and 90%, and then 5.80~8.70 g of acrylamide, 2.90~4.36 g of 2methyl-acrylamide-2methyl-propanesulfonic acid, 0.067~0.339 g of *N-N′*-methylene bis-acrylamide from Tianjin, China, and a certain amount of deionized water solution are added. The mixture is stirred, and reacted at room temperature for 1~2 h, soaked in distilled water, passed through a 10-mesh steel sieve, dried, and the water-absorbent resin is obtained.

### 2.2. Clod Preparation

The raw material soil is placed in an electric blast drying oven at a constant temperature, dried at a certain temperature for 12 h, cooled to room temperature, crushed by a pulverizer, and screened to remove sundries such as plant roots, stones, and debris. Deionized water was added to the dried soil according to 20% weight moisture content, sealed with a sample bag for 24 h, and pressed into 5 cm × 5 cm × 5 cm samples by a clod prototype according to 1.71 g/cm^3^. Natural drying conditions were applied to the pressed samples. US (unreinforced clod) represents the blank sample of the simulated soil block, and SAP (superabsorbent polymers) represents the simulated soil block sample reinforced with P (AA-AM-AMPS).

## 3. Measurements

### 3.1. Optimization of Synthesis Conditions

The water absorption of hydrogels is closely related to their slow-release properties. Therefore, the water absorption energy of P(AA-co-AM-co-AMPS) was used as a performance index for optimizing the synthesis conditions. The effects of three materials on the water absorption capacity of P(AA-co-AM-co-AMPS) were studied with different degrees of neutralization (ND = 50%, 60%, 70%, 80%, 90%), the dose of the crosslinking agent dosage (0~0.33 g), AA (m_AA_:m_AM_ = 1:5~10:5) AM dose (m_AM_:m_AMPS_ = 2:2~6:2), and the dose of AMPS (m _AMPS_: m _AM_ = 0~1) on the water absorption properties.

Based on the results of the above one-factor experiments, the optimal range of values was determined. The Box-Behnken model (BBD) in Response Surface Methodology (RSM) was used to code the above independent variables (X_1_, X_2_, and X_3_) according to Equation (1), with the parameter values and corresponding coded values shown in Table 1. Based on the BBD model, 17 experiments, including 5 sets of replicated experiments, were performed to evaluate the experimental errors, and the specific experimental parameters are shown in Table 2. The BBD model was utilized to derive the basic parameters provided by the analysis of variance (ANOVA), which were applied to evaluate the model’s significance and determine the optimal synthesis conditions.

**Table 1:** Independent variables and coded values for the BBD model.

Independent Variable Name	Code Name	Coded Value		
		−1	0	1
m (AA): m (MBA)	X_1_	32	40	48
m (AM): m (MBA)	X_2_	21	27	33
m (AMPS): m (MBA)	X_3_	11	13	15

(1) $$X_{i}={(x}_{i}-x_{0})/∆x\;\;i=1,\;2,\;3$$

$X_{i}$ is the coded value, $x_{i}$ is the true value of the independent variable, $x_{0}$ denotes the intermediate value of the independent variable, and ∆x denotes the absolute value of the difference between two neighboring variables.

(2) $$Y=\beta_{0}+\sum_{i=1}^{3}\beta_{i}X_{i}+\sum_{j=1}^{3}\beta_{\mathrm{ii}}X_{i}^{2}+\sum_{i=1}^{3}\sum_{j=i+1}^{3}\beta_{\mathrm{ij}}X_{i}X_{j}$$

Y is the response function, which represents the predicted value of the response; $\beta_{0}$ is a constant; $\beta_{i}$, $\beta_{ii}$, $\beta_{ij}$, represent the regression coefficients; X_i_ and X_j_ are the coded values of the independent variables.

**Table 2:** Experimental design of the BBD model.

Group	X_1_	X_2_	X_3_
1	48	32	13
2	40	32	10
3	48	26	10
4	32	26	10
5	40	21	16
6	32	21	13
7	40	26	13
8	40	26	13
9	40	26	13
10	40	21	10
11	40	26	13
12	40	26	13
13	48	21	13
14	48	26	16
15	32	26	16
16	32	32	13
17	40	32	16

### 3.2. Phase Analysis and Color Analysis

The chemical bonding of water-absorbent resins was examined by FT-IR (VERTEX 70, Bruker, Germany) to analyze the process of polymerization of monomers into polymers and to understand the mechanism of polymer production. The method is based on attenuated total reflection (ATR) spectroscopy with a wavelength range of 400–4000 cm^−1^ and a resolution of 4 cm^−1^. The XRD (TD3500) method was used to analyze the site and raw soil, while a colorimeter was used to analyze the reinforced soil.

### 3.3. Mechanics Performance Testing

A Shore A durometer from Wenzhou China was used to test the hardness of the surface of the sample, and the unconfined compressive strength of the sample was tested using a universal testing machine (1036PC).

### 3.4. Measurement of Water Absorption

The water-absorbing multiplicity of the resin is measured using the tea bag method. The mass of the high-absorbent resin, m_1_, is loaded into the tea bag, and the tea bag is immersed in deionized water. After ~4 h, when the resin reaches a dissolved state, cotton is used to absorb the water attached to the surface of the tea bag. The weight of the dissolved resin, m_2,_ is measured, then absorbent resin is poured out, and the mass of the tea bag, m_3_, is weighed. The water absorption capacity of high-absorbent resin Q (g/g) is calculated as follows:

(3) $$Q=\frac{m_{2}-m_{3}}{m_{1}}$$

## 4. Results and Discussion

**Figure 1:**
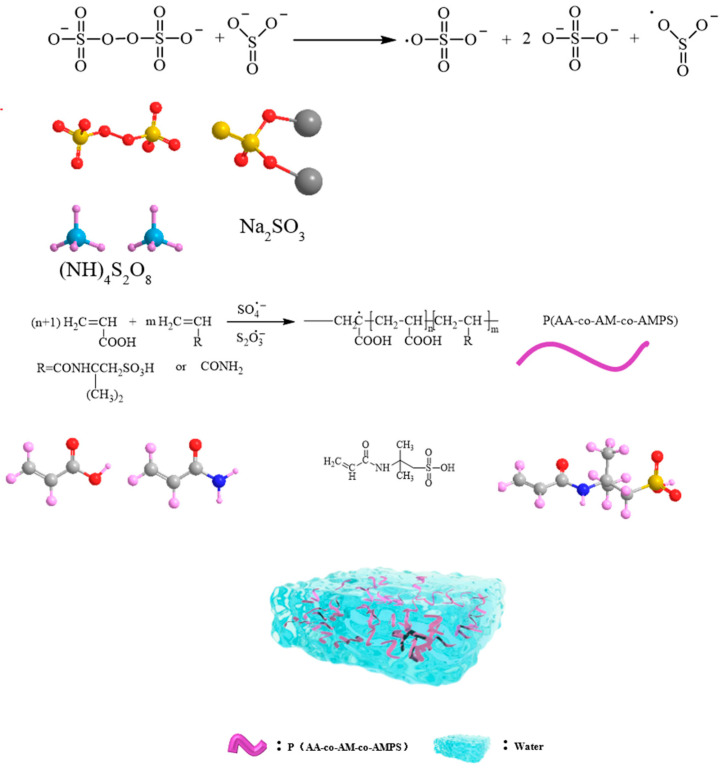
The mechanism diagram of P(AA-co-AM-co-AMPS).

**Figure 2:**
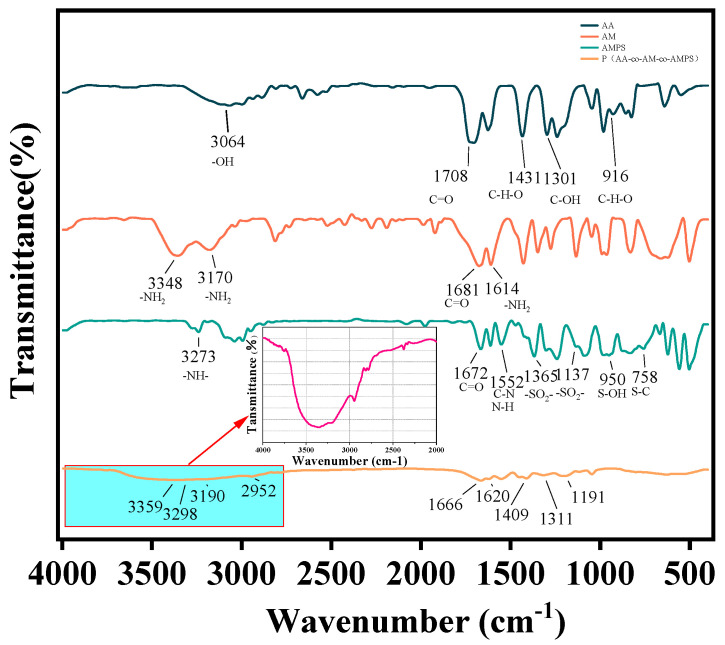
FT-IR spectra of P(AA-co-AM-co-AMPS).

The synthesis mechanism of P(AA-AM-AMPS) is shown in Figure 1. The FT-IR spectra of acrylic acid, as shown in Figure 2, exhibit characteristic peaks. In the AA spectrum, the peak at 3064 cm^−1^ corresponds to the OH stretching vibration of the carboxyl (COOH) group. The carboxylic acid carboxyl group forms a dimer due to hydrogen bonding, resulting in a strong OH stretching vibration that appears as a diffuse broadband in the range of 3200~2400 cm^−1^, forming a shoulder peak. The peak at 1708 cm^−1^ corresponds to the carboxylic acid carbonyl (C=O) stretching vibration. The dimeric state of the carboxylic acid, whether in the liquid or solid state, causes the stretching vibration frequency of the carbonyl group to range from 1710 to 1700 cm^−1^. Moreover, the peaks at 1431 cm^−1^ correspond to the carboxylic acid COOH C-H-O bending in-plane. The wave number 1301 cm^−1^ corresponds to the telescopic C-OH vibration of the carboxy (COOH) group, whereas, in carboxylic acids, the OH and carbonyl (C=O) atoms are directly connected. The lone pair of electrons in the orbitals of the O atoms and the π-electrons on the C=O form a P-π conjugation, increasing the density of the electron cloud between C-OH. Accordingly, the force constant of the C-OH telescopic vibration increases, thereby increasing the carboxylic acid C-OH telescopic vibrational frequency. The C-OH stretching vibration frequency of carboxylic acids is higher than that of alcohols, which generally range between 1310 cm^−1^ and 1250 cm^−1^. For the long-chain aliphatic dimeric carboxylic acids, this frequency is around 1300 cm^−1^. The peak at 916 cm^−1^ corresponds to the C-H-O out-of-plane bending vibration of the carboxyl (COOH) group. In the FT-IR spectrum of acrylamide, as shown in Figure 2 (AM mapping), the primary amide R-CONH_2_ molecule exhibits characteristic peaks. The variable angle vibration of NH_2_ is located at 1640–1600 cm^−1^ (1614 cm^−1^ in our case), and the C=O stretching vibration is located in the range of 1685–1660 cm^−1^ (1681 cm^−1^ in our case). The antisymmetric and symmetric stretching vibrations of NH_2_ have frequencies in the ranges of 3360–3320 cm^−1^ (3348 cm^−1^) and 3190–3160 cm^−1^ (3170 cm^−1^), respectively. The NH group stretching vibration in R-CO-NH-R occurs at 3273 cm^−1,^ within the range of 3320–3270 cm^−1^. The C=O bond stretching vibration occurs at 1672 cm^−1^, within the range of 1680–1630 cm^−1^. Moreover, the peak at 1552 cm^−1^ is the combined frequency peak of the C-N bond stretching and N-H bond bending vibrations.

The antisymmetric and symmetric stretching vibrations of SO_2_ in propane sulfonic acid (R-SO_2_-OH) are near 1340 cm^−1^ (1365 cm^−1^) and 1170 cm^−1^ (1137 cm^−1^), respectively, with strong absorption peaks. The S-OH and S-C stretching vibrations are near 900 cm^−1^ (950 cm^−1^) and 750 cm^−1^ (758 cm^−1^), respectively, with the former being stronger than the latter. 

The FT-IR spectrum of P(AA-co-AM-co-AMPS) is shown in Figure 2. The antisymmetric stretching vibration of COO occurs at 1620 cm^−1,^ and the symmetric stretching vibration of COO occurs at 1409 cm^−1^, indicating the presence of AA. The antisymmetric stretching vibration of NH_2_ occurs at 3359 cm^−1^. The symmetric stretching vibration of NH_2_ occurs at 3190 cm^−1^, and the C=O stretching vibration of the -CONH_2_ group occurs at 1666 cm^−1^, indicating the presence of AM. The stretching vibration of the NH group in R-CO-NH-R occurs at 3298 cm^−1^, The C=O bond stretching vibration occurs at 1666 cm^−1^, and the combined frequency peak at 1552 cm^−1^ corresponds to the C-N bond stretching vibration and the N-H bond bending vibration. The antisymmetric and symmetric SO_2_ stretching vibrations in propane sulfonic acid (R-SO_2_-OH) were observed at 1311 cm^−1^ and 1191 cm^−1^, respectively. Moreover, the NH stretching vibration of the secondary amine aliphatic appears as a spectral band in the range of 3290 cm^−1^ to 3270 cm^−1^ with weak intensity.

**Figure 3:**
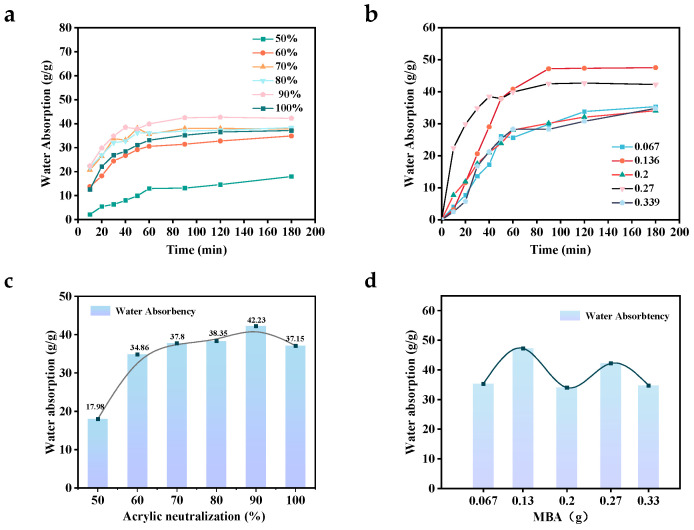
Plot of the multiplicity of water absorption P(AA-co-AM-co-AMPS).

The single-factor variable method was used to determine the degree of acrylic neutralization (ND) and the amount of crosslinker added. Figure 3a,c shows the effect of ND on the swelling properties of P(AA-co-AM-co-AMPS). From the figure, it can be seen that when the ND is 50%, the water absorption rate is 17.98 g/g, and the liquid absorption ability is poor. This is because when the value of ND is low, the acidity of the reaction system is too high, as is the polymerization reaction rate, which makes it easy to burst polymerization. As the ND value increases, the material swelling first increases and eventually declines. This is due to the increase in the ND value, which causes a decrease in the ability of the reaction system. This results in a slower polymerization rate and a decrease in the degree of self-crosslinking, which is conducive to the network structure of the crosslinking molding. At the same time, more -COOH groups deprotonate to -COO-, increasing the internal static repulsive force inside the network, which improves polymer network stretching and facilitates the water absorption process of the hydrogel. When the ND is too high, the side chain edges are long, blocking the voids in the network structure. Furthermore, the amount of Na+ contained inside the polymer network increases, occupying the binding sites of -COO-, which weakens the electrostatic force of repulsion. This leads to a decrease in the water absorption of the hydrogel.

The single-factor variable method was used to determine the degree of ND and the amount of crosslinker added. Figure 3a,c shows the effect of ND on the swelling properties of P(AA-co-AM-co-AMPS). From the figure, it can be seen that when the acrylic neutralization degree is 50%, the water absorption rate is 17.98 g/g, and the liquid absorption ability is poor. This is because when the ND value is low, the acidity of the reaction system is too high and the polymerization reaction rate is high, which makes it easy for polymerization to occur. With an increase in the ND value, the swelling of the material first increases and then decreases. This is because when the ND value increases, the acidity of the reaction system decreases, slowing down polymerization and decreasing the degree of self-crosslinking, which is beneficial to the network structure of the crosslinking of the model. Additionally, the more -COOH groups deprotonate to -COO-, increasing the internal static repulsive force inside the network and leading to polymer network stretching and aiding in the hydrogel’s water absorption. However, when the ND is too high, the side chain edges become long, blocking the network structure voids. Simultaneously, the amount of Na+ contained inside the polymer network increases, occupying the binding sites of -COO-, weakening the electrostatic repulsive force, and eventually reducing the hydrogel’s ability to absorb water.

Figure 3b,d illustrates the effect of the crosslinker content (MBA) on the swelling properties of P(AA-AM-AMPS). As shown in the figure, when the crosslinker content is less than 0.2 g, the water absorption of the gel increases and then decreases. This is because when the crosslinker content is low, the hydrogel network structure is loose and can accommodate a large number of water molecules. However, at this stage, the gel network structure is as loose as that of a soft gel, making it unsuitable for widespread applications. When the crosslinking agent content exceeds 0.2 g, the water absorption rate of the gel initially increases and then decreases. This is because the degree of crosslinking of the hydrogel increases with an increase in the content of the crosslinking agent, resulting in a stronger network structure that is less conducive to water molecule penetration, and the amount of water absorption is reduced.

**Figure 4:**
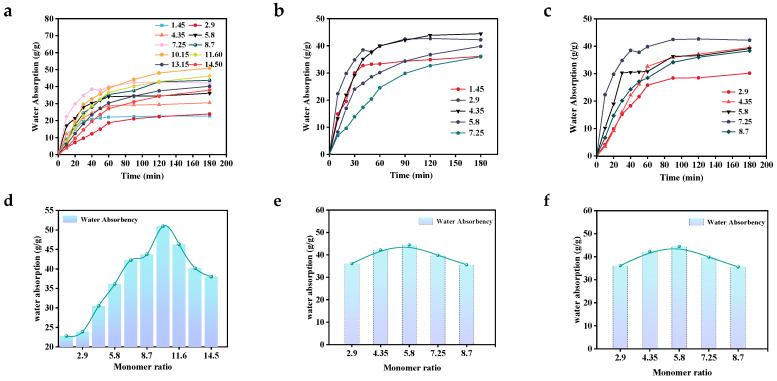
Effect of monomer content on the swelling properties of P (AA-AM-AMPS). (**a**) The effect of AA content on swelling performance graph (**b**) The effect of AM on swelling performance graph (**c**) The effect of AMPS on swelling performance graph (**d**) The effect of AA content on the water absorption performance of P(AA-AM-AMPS) graph (**e**) The effect of AM content on the water absorption performance of P(AA-AM-AMPS) graph (**f**) The effect of AMPS content on the water absorption performance of P(AA-AM-AMPS) graph.

Figure 4a,d,b,e show the effects of the dosage of acrylic acid (m _AA_: m _MBA_) and the dosage of acrylamide (m _AM_: m _MBA_) on the swelling properties of P(AA-AM-AMPS). As seen in Figure 4a,d, the maximum water absorption at the swelling equilibrium also reached its maximum value of 50.95 g/g when the m _AA_: m _MBA_ ratio increased from 1.45 to 10.15. The maximum water absorption measurement gradually decreased as the m _AA_: m _MBA_ ratio continued to increase. This is because the increase in the amount of AA introduces more hydrophilic groups (such as -OH and -COO-), which enhances the water-absorbing capacity of the gel. However, when the content of AA is further increased, the monomer in the reaction system becomes excessive, which is easy to produce the phenomenon of homopolymerization and bursting polymerization. The emergence of an acrylic acid homopolymer inside the gel network affects the internal structure of the water molecule network, causing the water-absorbing property to decrease rather than increase. When the ratio of m _AM_: m _MBA_ increases from 2.9 to 8.7, the water-absorbing capacity first increases and then decreases. This is because the addition of acrylamide introduces a hydrophilic group (-NH_2_), thereby enhancing the water-absorbing capacity. However, when too much acrylamide is added, it leads to polymerization, reducing the water-absorbing capacity. Acrylamide has strong adhesion, allowing it to easily adhere to the surface of the sample, which lays a solid foundation for its subsequent application.

Figure 4c,f shows the effect of 2-acrylamido-2-methyl-1-propanesulfonic acid on the swelling performance of P (AA-AM-AMPS). As shown in the figure, when the m _AMPS_: m _MBA_ ratio increased from 2.9 to 8.7, the water absorption capacity first increased and then decreased. This is attributed to the introduction of the sulfonic acid group (-SO_3_H), which initially enhanced the water absorption capacity. However, excessive addition of sulfonic acid group (-SO_3_H) led to homopolymerization, which led to a decrease in water absorption capacity. Nevertheless, since the additional amount of 2-acrylamido-2-methyl-1-propanesulfonic acid only accounts for 20% of the monomer content, the change in the water absorption capacity curve is minimal.

The effect of three factors at two levels on the water absorption performance of the materials was investigated using the dissolved water absorption rate of P(AA-co-AM-co-AMPS) as the response value. X_1_ = m _AA_: m _MAB_, X_2_ = m _AM_: m _MAB_, X_3_ = m _AMPS_: m _MAB_ were taken as independent variables, and the response value of the dissolved water absorption rate was measured after 17 sets of experiments, and the results are reported in Table 3. Through ANOVA, the F-value and *p*-value tests were performed, and the results are shown in Table 4. For the model as a whole and the primary and secondary variables, the smaller the *p*-value, the more significant the result. If the *p*-value is less than 0.05, it is considered significant, indicating that the model is well fitted or that the effect of a factor on the response value is significant. If the *p*-value is greater than 0.05, it indicates a poor fit to the predictive model or that the model is not significant. For the model used in this article, the F-value is greater than 0.05 and the *p*-value is less than 0.05; the fit term of 0.1052 is greater than 0.05, both of which indicate that the model is significant.

As shown in Table 4, the *p*-value of the primary term variable (X_1_) and the secondary term variables (X_1_X_3_, X_12_, X_22_ and X_32_) is less than 0.05, indicating that they have a significant effect on the hydrogel swelling properties. The *p*-value of the primary and the secondary term variables is greater than 0.05, indicating that their effects are not significant. Therefore, these are excluded from the fitted equations. From this, the equations for the real-valued quadratic polynomials are obtained as follows:

(4) $$Y=30.1959+0.599{3X}_{1}+0.1585X_{1}X_{3}-0.0270X_{1}^{2}-0.0004X_{2}^{2}-0.2489X_{3}^{2}$$

**Figure 5:**
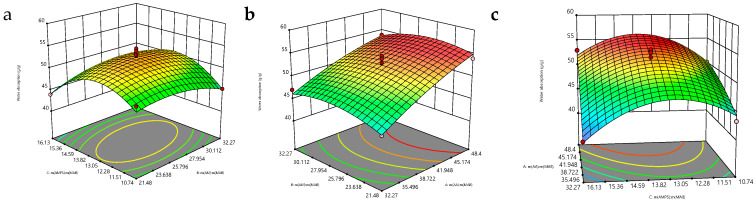
Surface plot of the surface response of the dissolved water absorption rate P(AA-co-AM-co-AMPS).

According to the BBD model, the swelling and water absorption rates of P(AA-AM-AMPS) are considered as the response values, and the response surface graph is plotted. The results are shown in Figure 5. Different factors can interact with each other on a response surface. In the response surface, the point located at the most convex position represents an optimal value. At this point, the material’s water absorption and swelling rates are the largest. In Figure 5a, the surface bends along two horizontal axes, forming a “claw” shape, and its bottom contour projection is a complete circle, which indicates that there is a strong interaction between the two independent factors, and an extreme point can be found in the middle of the simulated surface. In Figure 5b,c, the surface bends along one horizontal axis, which indicates that the interaction between the two factors is not as strong as that between the two independent factors. The interaction between the two factors in Figure 5b,c has less influence on the swelling property of the material. Finally, through model fitting, optimal preparation conditions can be derived as follows: m _AA_: m _MBA_ = 45.84, m _AM_: m _MBA_ = 22.54, and m _AMPS_: m _MBA_ = 14.68. This can be optimized by combining the results of previous single-variable method experiments as follows: ND = 90%, m _AA_: m _MBA_ = 45.84, m _AM_: m _MBA_ = 22.54, m _AMPS_: m _MBA_ = 14.68, m _MBA_ = 0.27 g.

**Figure 6:**
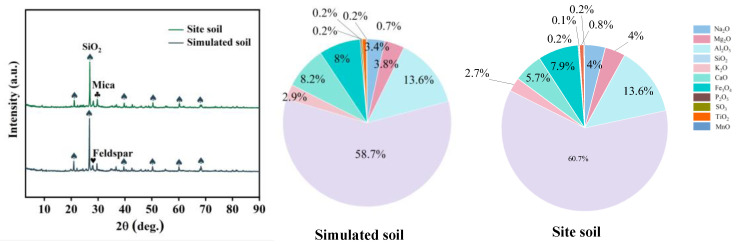
XRD and XRF spectra of the mausoleum of the Qin dynasty soil and raw material soil.

Figure 6 shows the XRD and XRF diagrams of the soil blocks. From the XRD diagrams, it can be seen that the raw material soil is similar in composition to the soil of the ruins, with SiO_2_, feldspar, and mica as the main components. Most elements in the soil blocks exist in the form of oxides. The calculation and analysis of the elemental results measured by XRF show that the percentages of Na_2_O, Mg_2_O, Al_2_O_3_, SiO_2_, K_2_O, CaO, Fe_3_O_4_, P_2_O_5_, SO_3_, Ti_3_O_2_, MnO in the soil of ruins are 3.4%, 3.8%, 13.6%, 58.7%, 2.9%, 8.2%, 8%, 0.2%, 0.2%, 0.7%, 0.2% and in the raw material soil are 4.2%, 4%, 13.6%, 60.7%, 2.7%, 5.7%, 7.9%, 0.2%, 0.1%, 0.8%, 0.2% respectively. The contents of the two soils are similar to each other; therefore, the raw material soil can be chosen to replace the soil of the ruins for subsequent experiments.

**Figure 7:**
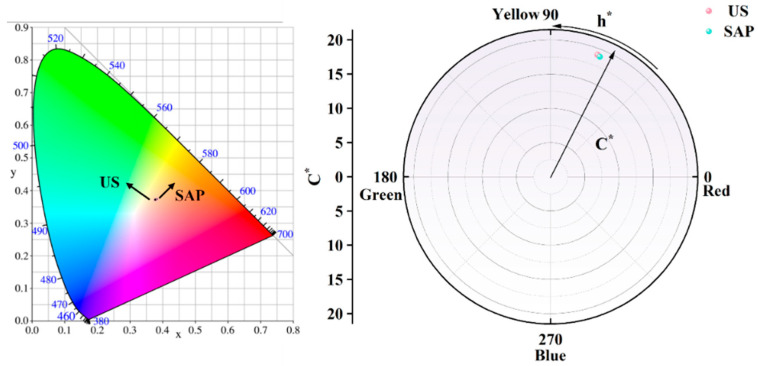
Chromaticity map of blank and SAP soil Chromaticity map of blank and SAP soil.

The color analysis of the soil blocks without and with SAP reinforcement is shown in Figure 7. From this figure, it can be observed that the saturation (C) and hue (h*) of the unreinforced soil blocks are slightly higher than those of the reinforced soil blocks. However, the chromatic aberration is not significant, and the colors are mainly distributed in the red and yellow zones. The analysis of the CIE plots shows that the colors of the samples fall at almost the same point, which follows the principle of reinforcing the soil ruins with a minimal difference in chromaticity. The color saturation and hue of the soil sample colors are closely related to the chemical composition of the soil, iron ion content, valence state, and internal microstructure of the soil.

As shown in Table 5, after 5 h of irradiation at an intensity of 0.5 W/m^2^, the unreinforced soil block exhibited noticeable cracks. This can be attributed to irradiation, which causes water loss within the soil, leading to contraction and uneven stress distribution. The unreinforced soil had a poor surface strength, and increasing the water content from spraying and subsequent irradiation caused irreversible damage to the soil, resulting in additional cracks. Conversely, the reinforced soil block with SAP showed only a few cracks. This improvement was due to the inclusion of SAP in the soil. When the soil’s internal water evaporates, the SAP releases the stored water, reducing the shrinkage volume of the soil. By reducing uneven stress throughout the drying process and preserving a more constant interior moisture level, this procedure supports in the prevention of crack growth. As a result, adding SAP significantly increases the soil’s ability to withstand radiation-induced cracking.

**Figure 8:**
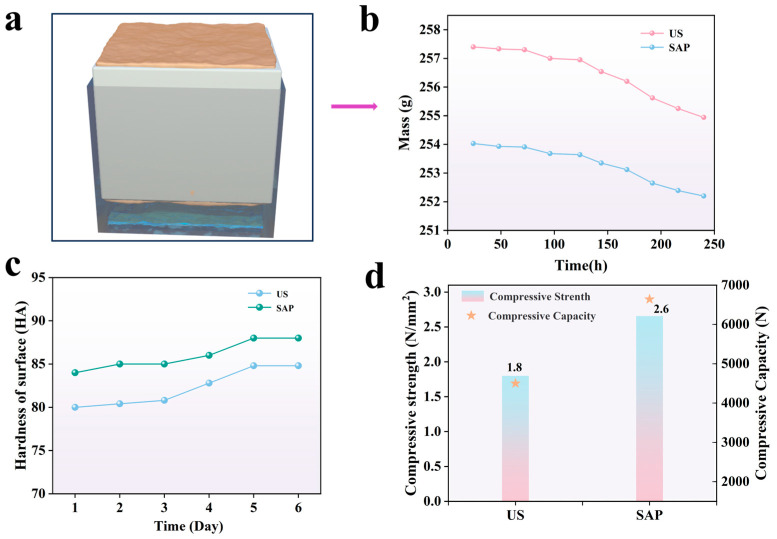
Graphs of changes in mechanical properties with curing time after sample treatment: (**a**) model, (**b**) air permeability, (**c**) surface hardness, and (**d**) compressive strength.

Figure 8a,b shows the air permeability test and the result graphs. During the experiment, the soil block was placed into the mold, wrapped with a raw cloth, and sealed with paraffin wax. Air permeability was observed on the upper and lower bottom surfaces. The weight of the block was measured, and the results are shown in Figure 8b. From Figure 8b, it can be seen that in the same interval of time, the mass loss in the unreinforced specimen was 0.03 g, while the mass loss in the specimen reinforced with SAP was 0.02 g, indicating a slight decrease in the quality of the sample. This is due to the addition of hydrogel, which fills the pores of the soil block, resulting in decreased air permeability. However, the slopes of air permeability for both specimens are the same. This is because, although the hydrogel fills the pores, it also has a honeycomb porous structure that increases the specific surface area of the pores. Consequently, the effect of the reinforcing agent on the air permeability of the soil is not significant, which is consistent with the principle of permeability for soil site reinforcement. Figure 8c shows the surface hardness curves of the samples. Over time, the surface hardness of both the blank sample and the reinforced sample increases until it remains unchanged. The surface hardness of the reinforced sample is slightly higher than that of the blank sample. This is due to the presence of water-absorbent resin in the reinforced sample, which has a much higher surface hardness compared to that of the blank sample. When the soil blocks are reinforced, the water-absorbent resin fills the gaps between the soil particles and improves the structure of the soil clods. This filling effect makes the clod dense and reduces the porosity inside the clod, thus improving its strength and surface hardness. Figure 8d compares the compressive strengths of the blank sample and the reinforced sample. The figure shows that the compressive strength of the reinforced sample is higher than that of the blank sample. This increase in compressive strength is most likely due to the hydrogel-filled soil pores, which improve water retention and air permeability and enhance the overall rigidity and load-bearing capability of the soil blocks.

## 5. Conclusions

In this paper, P(AA-AM-AMPS) was prepared by solution polymerization, and the synthesis scheme was optimized by an orthogonal experiment. The final scheme was applied as a reinforcement agent in the form of a stock solution of soil relics. Phase analysis of the water-absorbing resin and soil relics was conducted using FT-IR and X-ray diffraction (XRD). The aging resistance and compressive strength of the reinforced soil blocks were analyzed using a fluorescent ultraviolet (UV) aging chamber and universal testing machine. The experimental results showed that the optimal scheme for the preparation of P (AA-AM-AMPS) was ND = 90%, m _AA_: m _MBA_ = 45.84, m _AM_: m _MBA_ = 22.54, m _AMPS_: m _MBA_ = 14.68, m _MBA_ = 0.27 g. After aging resistance testing, the compressive strength of the soil blocks reinforced with the water-absorbing resin stock solution increased from the original 0.8 N/mm^2^ to 2.6 N/mm^2^, and the unconfined compressive strength decreased from the original 2.55 N/mm^2^ to 1.8 N/mm^2^. This indicates that the addition of a water-absorbing resin to the soil blocks exhibited excellent aging resistance and slightly improved compressive strength.

## Figures and Tables

**Table 3:** BBD experimental design with the response values of Q (g/g).

Group	X_1_	X_2_	X_3_	Q (g/g)
1	48	32	13	54
2	40	32	10	45
3	48	26	10	50
4	32	26	10	43
5	40	21	16	44
6	32	21	13	45
7	40	26	13	55
8	40	26	13	54
9	40	26	13	53
10	40	21	10	49
11	40	26	13	53
12	40	26	13	54
13	48	21	13	54
14	48	26	16	53
15	32	26	16	41
16	32	32	13	47
17	40	32	16	45

**Table 4:** BBD model ANOVA.

Source	Sum of Squared Deviations from the Mean(SS)	Degrees of Freedom (df)	Mean Square (MS)	F-Value	*p*-Value	
Model	344.42	9	38.27	31.14	<0.0001	significant
X_1_	154.09	1	154.09	125.38	<0.0001	
X_2_	0.0018	1	0.0018	0.0015	0.9705	
X_3_	2.82	1	2.82	2.29	0.1736	
X_1_ X_2_	1.30	1	1.30	1.06	0.3380	
X_1_ X_3_	7.98	1	7.98	6.49	0.0382	
X_2_ X_3_	5.86	1	5.86	4.77	0.0654	
X_1_^2^	8.57	1	8.57	6.97	0.0334	
X_2_^2^	22.93	1	22.93	18.66	0.0035	
X_3_^2^	128.59	1	128.59	104.63	<0.0001	
Residual	8.60	7	1.23			
Lack of Fit	6.47	3	2.16	4.05	0.1052	not significant
Pure Error	2.13	4	0.5330			

**Table 5:** Surface cracking of soil sites before and after aging.

Sample	US	SAP
**Before aging**	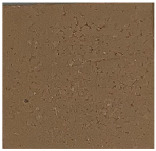	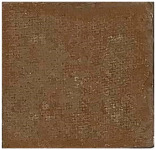
**After aging**	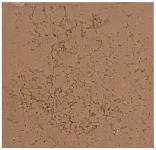	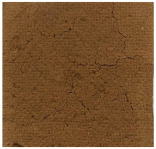

## Data Availability

The original contributions presented in this study are included in the article. Further inquiries can be directed to the corresponding author.
